# Supplementary material for: Refractory Salmonella Prosthetic Valve Endocarditis Complicated by Splenic Infarction and Aortic Pseudoaneurysm in a Patient with Double Prosthetic Valves: A Case Report
Source: Diagnostics (Basel). 2022 Aug 16;12(8):1982. doi: 10.3390/diagnostics12081982 (PMC9407200; doi:10.3390/diagnostics12081982)
Supplement: Supplementary file 1 [file diagnostics-12-01982-s001.zip › diagnostics-1864028-supplementary.pdf]

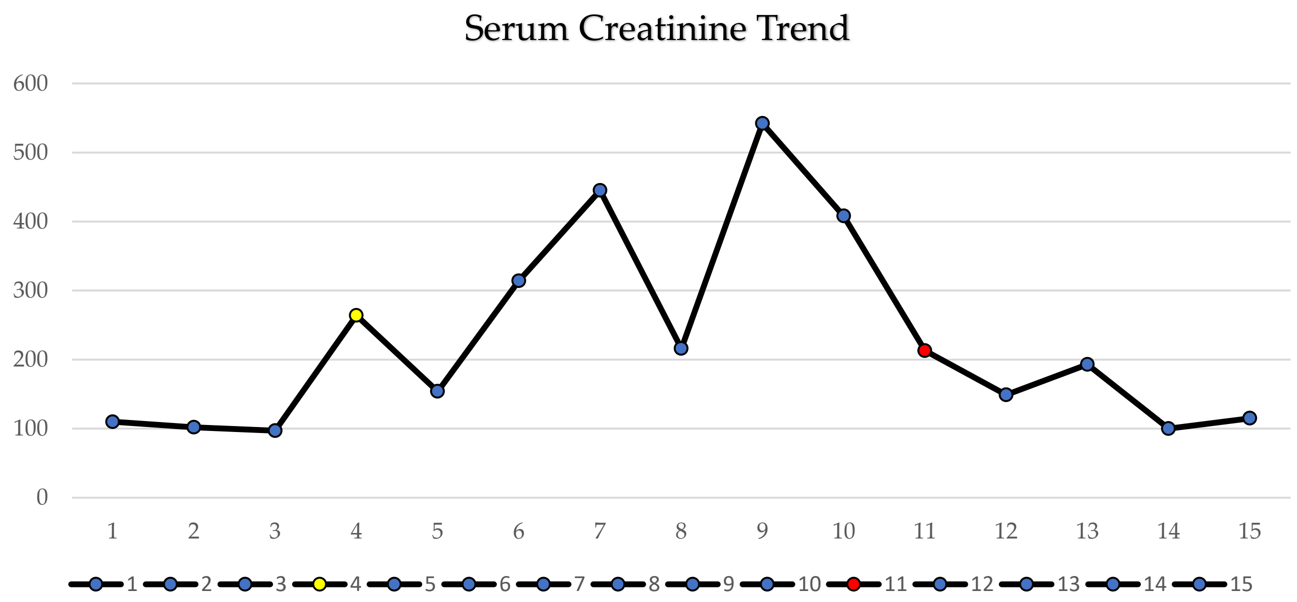

Figure S1. Trend of serum creatinine over a year and a half. The yellow dot indicates the time of admission and the red dot indicates the time of discharge.
